# Functional Analysis of RE1 Silencing Transcription Factor as a Putative Tumor Suppressor in Human Endometrial Cancer

**DOI:** 10.3390/ijms25179693

**Published:** 2024-09-07

**Authors:** Yasmin Abedin, Paige Minchella, Riley Peterson, Francesca Gonnella, Amanda Graham, Ian Cook, Melissa Javellana, Andrea Jewell, Lori Spoozak, Warren B. Nothnick

**Affiliations:** 1 Department of Obstetrics and Gynecology, University of Kansas Medical Center, Kansas City, KS 66160, USA; yabedin@kumc.edu (Y.A.); icook@kumc.edu (I.C.); mjavellana@kumc.edu (M.J.); ajewell@kumc.edu (A.J.); lspoozak@kumc.edu (L.S.); 2 Department of Cell Biology and Physiology, University of Kansas Medical Center, Kansas City, KS 66160, USA; pminchella@kumc.edu (P.M.); rpeterson6@kumc.edu (R.P.); fgonnella@unite.it (F.G.); agraham@kumc.edu (A.G.); 3 Department of Psychological Health and Territorial Sciences, School of Medicine and Health Sciences, “G. d’Annunzio” University of Chieti-Pescara, 66100 Chieti, Italy; 4 Unit of Molecular Genetics, Center for Advanced Studies and Technology (CAST), “G. d’Annunzio” University of Chieti-Pescara, 66100 Chieti, Italy; 5 Department of Bioscience and Technology for Food, Agriculture and Environment, University of Teramo, 64100 Teramo, Italy; 6 Department of Cancer Biology, University of Kansas Medical Center, Kansas City, KS 66160, USA; 7 Center for Reproductive Sciences, University of Kansas Medical Center, Kansas City, KS 66160, USA

**Keywords:** endometrial cancer, endometrioid sub-type, REST, MMP24, gene expression, protein expression, proliferation, migration, invasion

## Abstract

Uterine cancer is the most common gynecologic malignancy in the United States, with endometrioid endometrial adenocarcinoma (EC) being the most common histologic sub-type. Considering the molecular classifications of EC, efforts have been made to identify additional biomarkers that can assist in diagnosis, prognosis, and individualized therapy. We sought to explore the relationship of Repressor Element 1 (RE1) silencing transcription factor (REST), which downregulates neuronal genes in non-neuronal tissue, along with matrix metalloproteinase-24 (MMP24) and EC. We analyzed the expression of REST and MMP24 in 31 cases of endometrial cancer and 16 controls. We then explored the baseline expression of REST and MMP24 in two EC cell lines (Ishikawa and HEC-1-A) compared to a benign cell line (t-HESC) and subsequently evaluated proliferation, migration, and invasion in the setting of loss of *REST* gene expression. REST and MMP24 expression were significantly lower in human EC samples compared to control samples. REST was highly expressed in EC cell lines, but decreasing *REST* gene expression increased proliferation (FC: 1.13X, *p* < 0.0001), migration (1.72X, *p* < 0.0001), and invasion (FC: 7.77X, *p* < 0.05) in Ishikawa cells, which are hallmarks of cancer progression and metastasis. These findings elicit a potential role for REST as a putative tumor suppressor in EC.

## 1. Introduction

Uterine cancer is the most common gynecologic malignancy in the United States, with an estimated 66,200 new cancer cases and 13,030 deaths in 2023 [1]. The majority of uterine cancers include endometrial adenocarcinomas (ECs) and less than 10% include sarcomas [2]. Classically, EC has been categorized as Type I or II. Type I EC includes endometrioid EC, which is quite common and estrogen-driven. Type II EC includes serous, clear cell, and carcinosarcomas and is typically more aggressive and not estrogen-dependent [3,4]. This categorization allows for risk stratification based on histopathological features. Other histomorphologic features that are prognostic for survival and recurrence include grade, depth of myometrial invasion, the presence or absence of lymphovascular space invasion (LVSI), and tumor size [5,6].

More recently, EC has been risk stratified by its molecular classifications. The cancer genome atlas (TCGA) used whole-genome sequencing of EC tumors to identify four subgroups with distinct profiles in order to guide prognosis and treatment. These subgroups included DNA polymerase E (POLE, ultramutated), microsatellite instability (MSI-hypermutated), copy number low, and copy number high, ranging from good to poor prognosis, respectively [5,6,7,8]. POLE mutated tumors account for less than 10% of EC, lead to increased neoantigens and an improved immune response, and have a good prognosis [5,9,10]. The mismatch repair (MMR) pathway consists of specific DNA mismatch repair enzymes that are usually dependent on four genes including *MLH1*, *PMS2*, *MSH2*, and *MSH6*. Mutations in any of the genes can be hereditary (Lynch Syndrome) or somatic. Loss of function in any of the MMR proteins is termed dMMR and MSI-H refers to the phenotype that results from dMMR [5]. dMMR can be oncogenic, but also creates neoantigens that can make the tumors susceptible to immunotherapies such as monoclonal antibodies to programed death receptor 1 (PD-1), deeming it intermediate in prognosis. The copy number low classification is the most common classification and refers to the endometrioid-like ECs without a POLE mutation, abnormal tumor protein 53 (TP53), or dMMR/MSI-H and has intermediate prognosis. Copy number high refers to the group with serous-like EC histology and evaluation includes over 1.3 million single-nucleotide polymorphisms to determine the copy number status. Patients with copy number high EC have the poorest prognosis out of the four subgroups and most have a *TP53* mutation [5]. Although these classifications exist, not all institutions have adopted molecular tumor testing [6,8]. Immunohistochemistry (IHC) has been used to augment the characterization of tumors, specifically by examining estrogen receptor (ER) and progesterone receptor (PR) percentage/status, the presence or absence of MMR proteins, and abnormal TP53 to help distinguish EC sub-types and direct treatment [5,6,8,11].

Considering the molecular classifications and immunohistochemistry (IHC), efforts have been made to identify additional biomarkers that can assist in the diagnosis, prognosis, and individualized therapy for EC [11]. One potential biomarker is Repressor Element 1 (RE1) silencing transcription factor (REST), which is a transcription factor that downregulates neuronal genes in the nucleus in non-neuronal tissue [12,13,14,15]. REST has been linked to several cancers, including colon, small cell lung, and breast cancer as a putative tumor suppressor and decreased expression of REST may lead to cancer [15,16,17,18]. In particular, loss of REST played a role in the pathogenesis of a subset of breast cancers through the activation of the ER signaling pathway [18].

REST has not been previously studied in EC. However, REST has been investigated in other gynecologic malignancies with inconsistent findings, showing both oncogenic and tumor suppressor properties. In ovarian cancer (OvCa) cells, REST expression was lower in a chemotherapy-resistant OvCa cell line compared to a chemotherapy-sensitive cell line [19]. Further, knockdown of *REST* in other OvCA cell lines led to decreased proliferation, indicating the potential role of an oncogene [20]. In cervical cancer, REST expression was significantly lower in cancer samples compared to benign samples, suggesting a possible function as a tumor suppressor [21]. Given these mixed results in other gynecologic malignancies, the investigation of the function of REST in EC becomes essential to understand its potential impact.

Additional markers of interest include those that may play a role in the epithelial to mesenchymal transition (EMT), which is required for cancer progression through proliferation, migration, and invasion [22,23,24]. Matrix metalloproteinases (MMPs) have been linked to the EMT process through breakdown of the extracellular matrix (ECM), leading to tumor invasion and metastasis [24]. One such MMP is MMP24, also known as membrane type 5-MMP, that can activate other MMPs and has been linked to REST [18,24,25,26].

Given that the relationship between REST and EC has not been previously studied, and considering the apparent connection between REST and estrogen, we found it intriguing to investigate the role of REST in endometrioid EC. Our objective was to determine the relationship between REST and EC, while also investigating MMP24. We hypothesized that REST expression would be decreased in EC tissue and cell lines and that REST deficiency would contribute to the hallmarks of tumor progression and spread. We first determined endogenous expression of REST and MMP24 in endometrioid EC patient tissue samples compared to benign tissue samples. We then explored the relationship between REST and MMP24 in addition to the association of REST expression with patient and tumor characteristics. We then determined the baseline expression of REST and MMP24 in EC cell lines compared to a benign cell line and subsequently evaluated proliferation, migration, and invasion in the setting of loss of *REST* gene expression. This study will help elucidate the potential role of REST as a prognostic marker and in the pathogenesis of EC.

## 2. Results

### 2.1. REST Expression Is Decreased in EC Compared to Controls

Baseline patient demographics for controls (N = 16), early-stage EC (N = 15), and advanced-stage EC (N = 16) are listed in Table 1. There were no differences in any variables among the three groups.

Tumor characteristics are listed in Table 2. There was a statistically significant difference in grade, with early stage consisting of mostly grade 1 tumors (73.3%) and advanced stage having mostly grade 2 tumors (50.0%).

**Figure 1:**
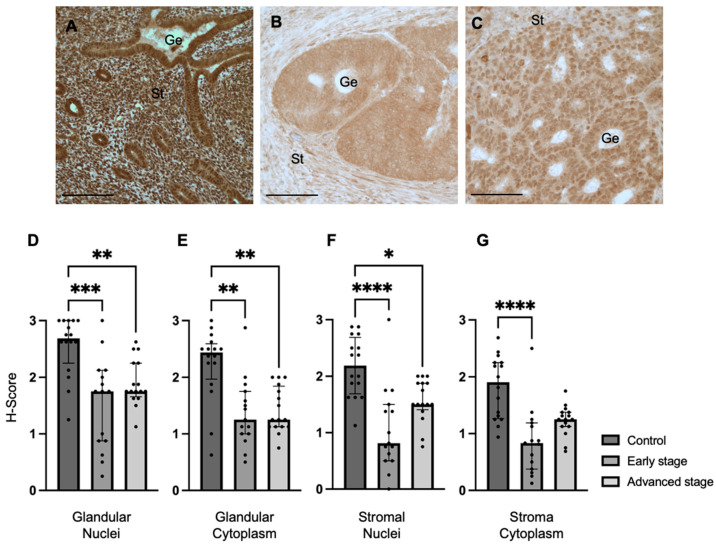
**REST expression in control and EC specimens.** (**A**–**C**) Representative images of REST expression in control (N = 16), early-stage (N = 15), and advanced-stage EC (N = 16) localized to the nucleus and cytoplasm of the glandular epithelium (Ge) and stroma (St). REST expression was decreased in early-stage and advanced-stage EC specimens compared to controls in the (**D**) epithelial glandular nuclei, (**E**) glandular cytoplasm, and (**F**) stromal nuclei. REST expression was only decreased in early-stage EC compared to controls in the stromal cytoplasm (**G**). Data are represented as median with IQR, * *p* < 0.05, ** *p* < 0.01, *** *p* < 0.001, **** *p* < 0.0001. Scale bars are 100 µm.

We determined REST expression in the endometrial epithelial glands and stroma of control specimens compared to early-stage and advanced-stage EC specimens by IHC (Figure 1A–C). REST expression was decreased in the glandular epithelial cell nuclei of early-stage EC specimens (median H-score: 1.75 versus 2.69, *p* < 0.001) and advanced-stage EC specimens (median H-score 1.75 versus 2.69, *p* < 0.01) compared to controls (Figure 1D). REST expression was also decreased in the glandular cytoplasm of early-stage (median H-score: 1.25 versus 2.44, *p* < 0.01) and advanced-stage (median H-score: 1.25 versus 2.44, *p* < 0.01) EC specimens compared to controls (Figure 1E). Similarly, REST expression was decreased in the stromal nuclei of both-early stage (median H-score: 0.81 versus 2.19, *p* < 0.0001) and advanced-stage (median H-score 1.50 versus 2.19, *p* < 0.05) EC samples compared to controls (Figure 1F). REST was only decreased in early-stage EC samples compared to controls in the stromal cytoplasm (median H-score: 0.83 versus 1.91, *p* < 0.0001, Figure 1G).

Because REST is proposed to function as a gene repressor at the level of the cell nucleus, we further explored the relationship between REST expression in the glandular nuclei and patient characteristics including stage of cancer, age, BMI, race, medications for comorbidities including anti-diabetics, hypertensives, and lipids, and smoking and alcohol use history. REST expression in the nuclei of glands had an association with early-stage EC (β-coefficient: −1.19; 95% CI: −1.64 to −0.73, *p* < 0.01), advanced-stage EC (β-coefficient: −0.83; 95% CI: −1.31 to −0.34, *p* < 0.01) and age (β-coefficient: 0.03; 95% CI: 0.01 to 0.04, *p* < 0.01). The other variables did not impact REST expression in the glandular nuclei.

When examining tumor characteristics and REST expression, there was a statistically significant negative correlation between REST expression in the glandular nuclei and ER percentage in the tumor (Spearman r: −0.44, *p* = 0.01). There were no associations found between REST expression in any of the other localizations and tumor characteristics such as grade, presence of LVSI, PR percentage, presence or absence of abnormal TP53, and MMR status.

### 2.2. MMP24 Expression Is Decreased in EC Compared to Controls

**Figure 2:**
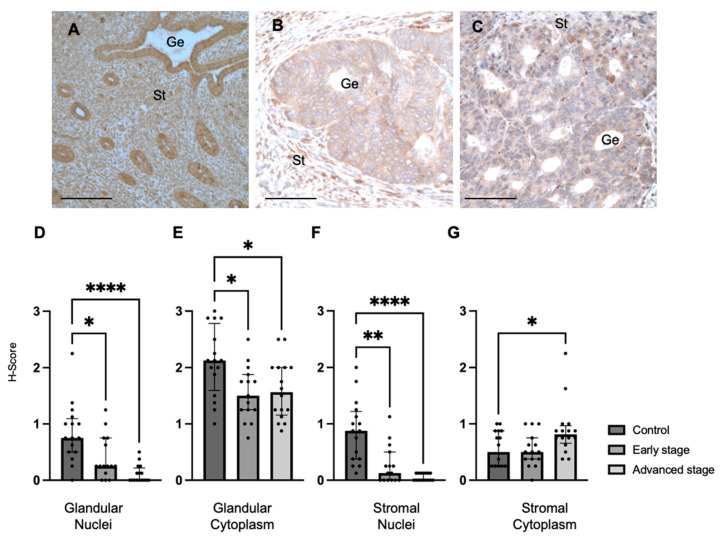
**MMP24 expression in control and EC specimens.** (**A**–**C**) Representative images of MMP24 expression in control (N = 16), early-stage (N = 15), and advanced-stage EC (N = 16) localized to the nucleus and cytoplasm of the glandular epithelium (Ge) and stroma (St). MMP24 expression was decreased in early-stage and advanced-stage EC specimens compared to controls in the (**D**) epithelial glandular nuclei, (**E**) glandular cytoplasm, and (**F**) stromal nuclei. (**G**) MMP24 expression was only increased in advanced-stage EC compared to controls in the stromal cytoplasm. Data are represented as median with IQR, * *p* < 0.05, ** *p* < 0.01, **** *p* < 0.0001. Scale bars are 100 µm.

We also determined MMP24 expression in the endometrial glands and stroma of control specimens compared to early-stage and advanced-stage EC specimens by IHC (Figure 2A–C). We found that MMP24 expression was decreased in the glandular nuclei of early-stage (median H-score: 0.25 versus 0.75, *p* < 0.05) and advanced-stage (median H-score 0.00 versus 0.75, *p* < 0.0001) EC specimens compared to control specimens (Figure 2D). MMP24 expression was also decreased in the glandular cytoplasm of early-stage (median H-score: 1.50 versus 2.13, *p* < 0.05) and advanced-stage (median H-score: 1.56 versus 2.13, *p* < 0.05) EC specimens compared to controls (Figure 2E). Similarly, compared to controls, MMP24 expression was decreased in the stromal nuclei of both early-stage (median H-score: 0.13 versus 0.88, *p* < 0.01) and advanced-stage (median H-score 0.00 versus 0.88 *p* < 0.0001) EC samples (Figure 2F). MMP24 expression was increased the stromal cytoplasm of advanced-stage EC compared to controls (median H-score: 0.81 versus 0.50, *p* < 0.05, Figure 2G).

### 2.3. REST and MMP24 Relationship

**Figure 3:**
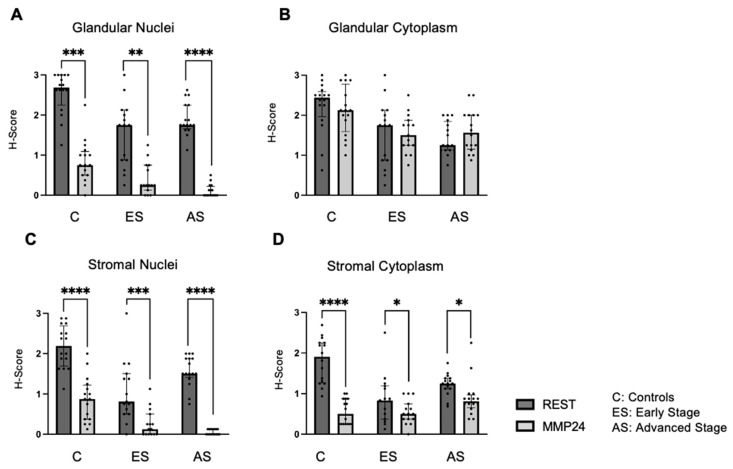
**Comparison of REST and MMP24 expression in each cellular localization.** REST and MMP24 expression were compared in each cellular localization for controls, early-stage, and advanced-stage EC samples. MMP24 expression was decreased compared to REST expression in the (**A**) epithelial glandular nuclei, (**C**) stromal nuclei, and (**D**) stromal cytoplasm for control, early-stage, and advanced-stage EC samples. There was no difference in MMP24 expression compared to REST expression in the glandular cytoplasm (**B**). Data are represented as median with IQR, * *p* < 0.05, ** *p* < 0.01, *** *p* < 0.001,**** *p* < 0.0001.

The relationship between REST and MMP24 expression was explored in each of the cellular localizations for control (C), early-stage (ES), and advanced-stage (AS) EC samples (Figure 3). MMP24 expression levels were lower than REST expression levels in the glandular nuclei (Figure 3A) for controls (*p* < 0.001), early-stage (*p* < 0.01) and advanced-stage specimens (*p* < 0.0001). MMP24 expression was also decreased compared to REST expression levels in the stromal nuclei and stromal cytoplasm (Figure 3C,D). There was a trend towards decreased MMP24 and REST expression levels in the glandular cytoplasm of specimens; however, it was not statistically significant (Figure 3B).

**Figure 4:**
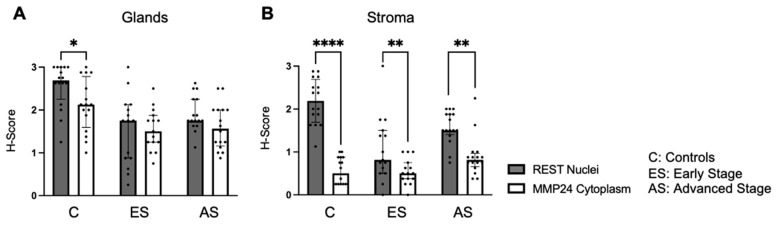
**Comparison of nuclear REST to cytoplasmic MMP24 expression in glands and stroma.** (**A**) When comparing REST nuclear expression to MMP24 cytoplasmic expression within the glands, there was a significant decrease in MMP24 expression only in the control samples, but not in the early-stage or advanced-stage samples. (**B**) There was a decrease in MMP24 cytoplasmic expression compared to REST nuclear expression in the stroma of controls, early-stage EC, and advanced-stage EC specimens. Data are represented as median with IQR, * *p* < 0.05, ** *p* < 0.01, **** *p* < 0.0001.

Further, nuclear REST expression was compared to cytoplasmic MMP24 expression in the glandular epithelium and stroma (Figure 4). There was decreased MMP24 expression in the cytoplasm compared to the nuclear REST expression in the glands of control specimens (*p* < 0.05), but not in the cancer specimens (Figure 4A). There was a decrease in the cytoplasmic MMP24 expression compared to the nuclear REST expression in the stroma of control (*p* < 0.0001), early-stage EC (*p* < 0.01), and advanced-stage EC (*p* < 0.01) specimens (Figure 4B).

### 2.4. REST Expression Is Increased in EC Cell Lines

**Figure 5:**
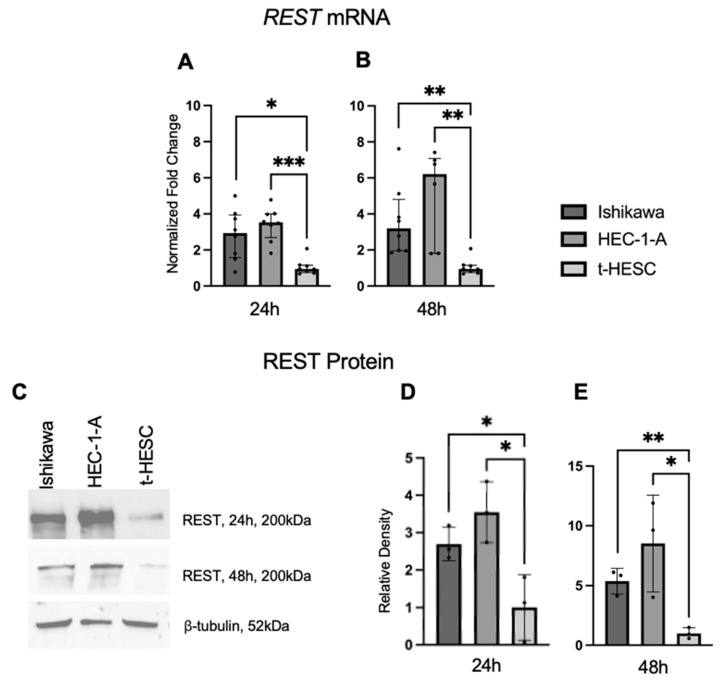
**REST expression in EC cell lines**. Baseline *REST* expression was increased in Ishikawa and HEC-1-A compared to t-HESC at 24 h (**A**) and 48 h (**B**). Data are represented as median with IQR. (**C**) Representative Western blot images of REST expression for cell lines. Baseline REST protein expression was increased in Ishikawa and HEC-1-A compared to t-HESC at 24 h (**D**) and 48 h (**E**). Data are represented as mean with SD, * *p* < 0.05, ** *p* < 0.01, *** *p* < 0.001.

REST gene and protein expression were characterized in a benign cell line, t-HESC, and two EC cell lines, Ishikawa and HEC-1-A. Using qRT-PCR, we found that baseline *REST* mRNA expression was higher in the Ishikawa (FC: 3.09X, *p* < 0.05; FC: 3.37X, *p* < 0.01) and HEC-1-A (FC: 3.72X, *p* < 0.01; FC: 6.54X, *p* < 0.01) cell lines compared to t-HESC at 24 and 48 h, respectively (Figure 5A,B). Baseline REST protein expression by Western blot (Figure 5C) was higher in Ishikawa (FC: 2.70X, *p* < 0.05; FC: 5.36, *p* < 0.01) and HEC-1-A (FC: 3.55X, *p* < 0.05; FC: 8.52X, *p* < 0.05) compared to t-HESC at 24 and 48 h, respectively (Figure 5D,E). Full Western blot images can be found in Supplementary Materials.

### 2.5. MMP24 Is Differentially Expressed in EC Cell Lines

**Figure 6:**
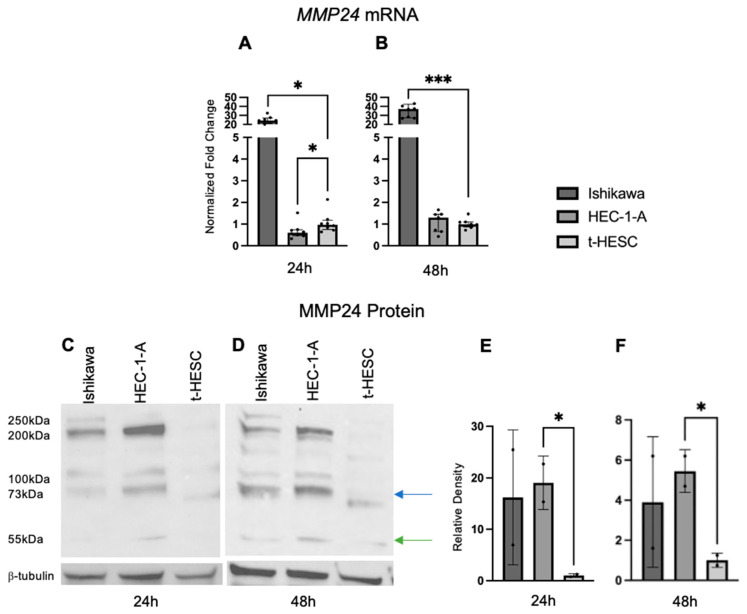
**MMP24 expression in EC cell lines**. (**A**,**B**) Baseline *MMP24* gene expression was increased in Ishikawa compared to t-HESC at 24 h and 48 h. *MMP24* was decreased in HEC-1-A compared to t-HESC only at 24 h. Data are represented as median with IQR. (**C**,**D**) Representative Western blot images of MMP24 expression with multiple bands at 24 h and 48 h. MMP24 can be detected at 73 kDa (blue arrow) as the full-sized band and 55kDa (green arrow) as the active band. Densitometry for the active band showed increased MMP24 expression in HEC-1-A compared to t-HESC at 24 h (**E**) and 48 h (**F**). Data are represented as mean with SD, * *p* < 0.05, *** *p* < 0.001.

Baseline *MMP24* gene expression was also explored in EC cell lines and t-HESC (Figure 6A,B). In Ishikawa cells, *MMP24* levels were higher (FC: 24.90X, *p* < 0.001; FC: 37.60X, *p* < 0.001) compared to those of t-HESC at 24 and 48 h, respectively. In HEC-1-A, *MMP24* levels were lower (FC: 0.62X, *p* < 0.05) than those of t-HESC at 24 h, but there was no difference at 48 h. Western blot for MMP24 demonstrated multiple bands with similar patterns in each cell line at 24 and 48 h (Figure 6C,D); the band detected at 73kDa (blue arrow) is the full-sized band, but the active form can be detected around 55kDa (green arrow) [24]. Densitometry of the active band revealed that endogenous MMP24 protein expression trended higher in Ishikawa than in t-HESC at 24 and 48 h, but not significantly. Active MMP24 protein levels were statistically higher in HEC-1-A compared to t-HESC at 24 h (FC: 19.03X, *p* < 0.05) and 48 h (FC: 5.45X, *p* < 0.05) (Figure 6E,F). Full Western blot images can be found in Supplementary Materials. 

### 2.6. Knockdown of REST Expression Increases Proliferation, Migration, and Invasion in Ishikawa Cells

Because of the high *REST* gene expression and REST protein levels in the cancer cell lines, *REST* expression was decreased using reverse transfection and the following functional assays were carried out.

**Figure 7:**
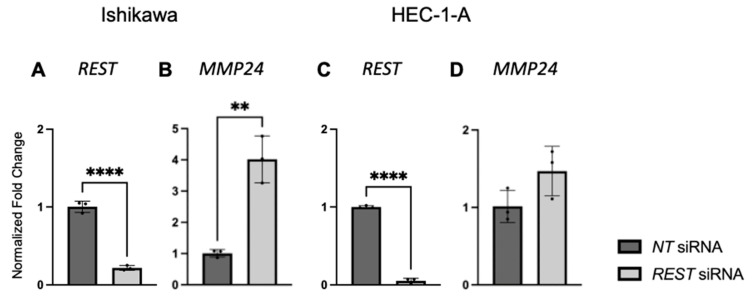
*REST* and *MMP24* gene expression after *REST* knockdown in EC cell lines. After double transfection using *NT* and *REST* siRNA, *REST* was decreased (**A**) and *MMP24* was increased (**B**) in Ishikawa cells. In HEC-1-A, *REST* was decreased (**C**) but there was no change in MMP24 (**D**) after the double transfection. Data are represented as mean with SD, ** *p* < 0.01, **** *p* < 0.0001.

After EC cell lines were double transfected with *NT* or *REST* siRNA, decreased *REST* levels were confirmed using qRT-PCR. In Ishikawa cells, when *REST* levels were decreased, *MMP24* levels were increased in the *REST* siRNA samples compared to the *NT* samples (Figure 7A,B). In HEC-1-A, when *REST* was decreased, there was no change in *MMP24* levels between *NT* and *REST* siRNA samples (Figure 7C,D).

**Figure 8:**
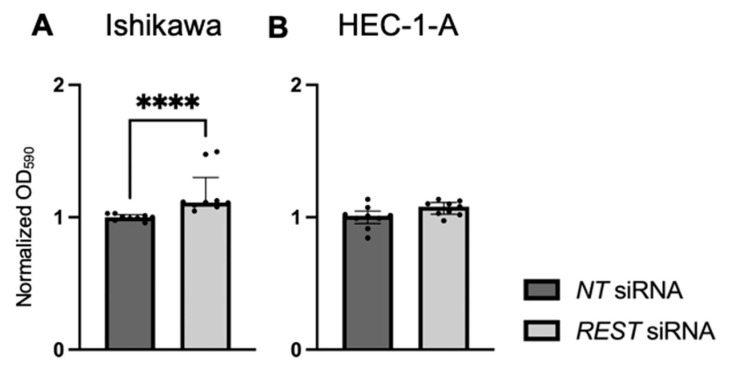
Impact of *REST* knockdown on EC cell line proliferation. (**A**) There was increased proliferation in Ishikawa cells by 1.13-fold after *REST* was knocked down, represented by normalized OD590 values. (**B**) There was no change in proliferation in HEC-1-A after *REST* was knocked down. Data are represented as median with IQR, **** *p* < 0.0001.

Cellular proliferation was assessed using a crystal violet assay and normalized OD at 590 nm values were reported. There was increased cell viability in Ishikawa cells when *REST* was knocked down (Figure 8A; FC: 1.13X, *p* < 0.0001). There was no change in cell viability in HEC-1-A cells (Figure 8B).

**Figure 9:**
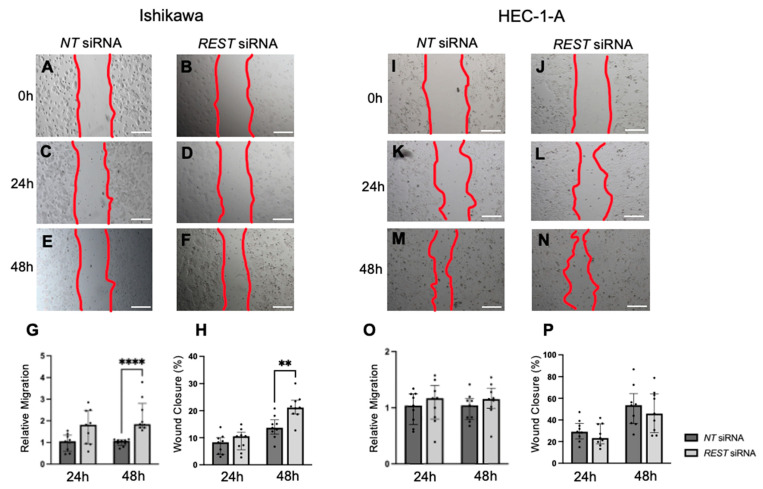
**Impact of *REST* knockdown on EC cell migration.** Representative images for scratch assay in Ishikawa cells at 0 h (**A**,**B**), 24 h (**C**,**D**), and 48 h (**E**,**F**). (**G**) There was a statistically significant increase in relative migration by 1.72-fold in Ishikawa cells after 48 h of *REST* knockdown. (**H**) There was also a significant increase in percent wound closure from 13.7% to 21.2% after 48 h. Representative images for scratch assay in HEC-1-A cells at 0 h (**I**,**J**), 24 h (**K**,**L**), and 48 h (**M**,**N**). (**O**,**P**) There were no changes in relative migration or percent wound closure in HEC-1-A cells. Data are represented as median with IQR, ** *p* < 0.01, **** *p* < 0.0001. Scale bars are 500 µm.

Migration was also assessed using a scratch assay and the area within the scratch was measured at 0, 24, and 48 h and normalized. In Ishikawa cells, there was an increase in relative migration after 48 h in the *REST* siRNA knocked down samples compared to the *NT* siRNA samples (Figure 9A–G; FC: 1.72X, *p* < 0.0001); however, there was no difference at 24 h. Similarly, there was a statistically significant increase in percent wound closure from 13.7% to 21.2% after 48 h in Ishikawa cells (Figure 9H). There was no change in migration or percent wound closure in HEC-1-A (Figure 9I–P).

**Figure 10:**
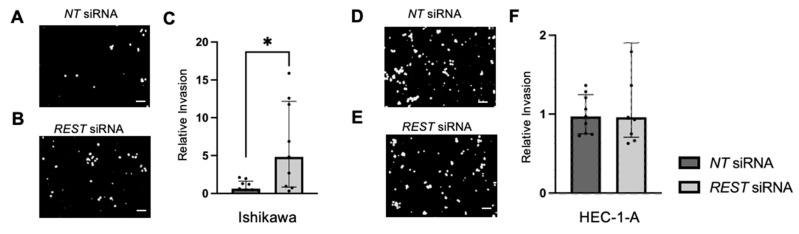
Impact of *REST* knockdown on EC cell invasion. Representative images of the DAPI-stained (in black and white) invading cells for the trans-well invasion assay for Ishikawa cells (**A**,**B**) and HEC-1-A cells (**D**,**E**). (**C**) There was a statistically significant increase in relative invasion by 7.77-fold in Ishikawa cells. (**F**) There was no change in relative invasion in HEC-1-A cells. Data are represented as median with IQR, * *p* < 0.05. Scale bars are 100 µm.

Trans-well invasion assays were conducted and the percent invasion was measured and normalized. Representative images of the DAPI stained undersurface of the membrane are depicted for Ishikawa cells (Figure 10A,B) and HEC-1-A cells (Figure 10D,E). In Ishikawa, there was a significant increase in relative invasion (FC: 7.77X, *p* < 0.05) in the *REST* siRNA knocked down samples compared to the *NT* siRNA samples (Figure 10C). There was no change in relative invasion in HEC-1-A cells (Figure 10F).

## 3. Discussion

In order to determine if REST can be used as a potential prognostic biomarker, we investigated the expression of REST in endometrioid EC patient tissue and EC cell lines. We demonstrated that REST expression was decreased in EC patient samples compared to control samples; however, there was no difference in expression between early- and advanced-stage EC. Conversely, REST expression was increased in EC cell lines compared to a benign cell line. But, when *REST* gene expression was decreased in the EC cell lines, the cell line that most closely resembles endometrioid EC, Ishikawa, demonstrated increased aggressiveness through proliferation, migration, and invasion, indicating a potential role for the loss of *REST* in the pathogenesis of endometrioid EC. Expanding on the difference in REST expression between EC and control samples, and the aggressive nature that the loss of REST manifests, may help determine the role of REST as a potential prognostic marker and putative tumor suppressor.

Because REST is proposed to mediate regulation of gene expression which occurs in the nucleus, we investigated the relationship between REST expression in the glandular nuclei and patient demographics. After controlling for age, BMI, and comorbidities, early- and advanced-stage EC were independently associated with REST expression in the glandular nuclei, again demonstrating that decreased REST levels may impact the pathogenesis of EC. Older age was also associated with higher REST levels, which is counterintuitive because EC is generally diagnosed at older ages and REST expression was lower in the EC samples [1,2]. However, this may be explained by the limited sample size and similarity in age range among the patient groups.

We also examined the relationship between REST expression in the EC samples and tumor characteristics, such as grade, presence of LVSI, ER/PR percentage, presence or absence of abnormal TP53, and MMR status. We found an inverse relationship between REST expression in the glandular nuclei of EC samples and ER percentage; that is, low REST scores correlated with high ER percentage. Similarly, prior studies have indicated a relationship between REST and estrogen signaling in breast cancer cell lines and in REST conditional knockout mice with uterine pathology, such as leiomyomas, which support our findings [18,26]. Additionally, the majority of endometrioid ECs are estrogen-driven and may have the molecular classification of copy number low [3,5]; thus, recurrence therapy often includes hormone therapy with aromatase inhibitors or selective estrogen receptor modulators [27,28]. Because of this, REST may be a biomarker that is applicable to the majority of ECs. In order to further elicit REST as a prognostic marker, future translational studies may involve investigating REST expression level and response to hormone therapy in the recurrent setting.

Endogenous *REST* gene expression and REST protein expression was higher in two EC cell lines, Ishikawa (Type I EC) and HEC-1-A (Type II EC), compared to a benign cell line, t-HESC. This was counter to our findings in the human endometrial samples. A similar inconsistency was seen in cervical cancer. REST expression in human cervical squamous cell carcinoma (SCC) samples was lower than that of control samples by IHC; however, cervical SCC cell lines showed no statistical difference in REST expression compared to a benign cell line [21]. The difference in results between human studies and cell line studies could have been observed for several reasons. Endometrial tissue is morphologically heterogeneous, whereas immortalized cell lines represent one cell type. Further, immortalized cell lines undergo processes that impair cell cycle checkpoint pathways, over-activation of the telomerase enzyme, or upregulation of oncogenes or oncoproteins that lead to the ability to infinitely divide [29]. Also, two-dimensional mono-cell culture may not replicate cell-to-cell and cell-to-extracellular environment interactions that are present in human tissues or tumors [30]. These features of immortalized cell lines may contribute to the difference in REST expression that is seen between EC human samples and cell lines. Further studies involving three-dimensional or organoid cell culture may be useful. Nevertheless, when *REST* expression was decreased by siRNA transfection in our cell lines, there was an increase in proliferation, migration, and invasion in Ishikawa cells, the cell line that most closely resembles endometrioid EC. There was no change in proliferation, migration, or invasion in HEC-1-A, the cell line that more closely resembles serous histology or Type II EC [31]. The differences in functional assays between the EC cell lines when *REST* was decreased may indicate its role in the pathogenesis of endometrioid EC and not more aggressive sub-types.

The expression of MMP24 in EC patient tissue and EC cell lines was also determined. We found that MMP24 expression was generally decreased in EC patient samples compared to control samples in most cellular compartments. Although MMPs contribute to ECM breakdown and tumor progression, elevated MMP24 levels were not seen in the human cancer tissue. This may be due to a more prominent role for MMP24 during the early pathogenesis of EC, but not after it has already developed, as individual MMPs are involved in varying degrees in the different stages of cancer progression by activating other MMPs or degrading ECM components [23,24,25,32]. However, MMP24 was elevated in the stromal cytoplasm of advanced-stage EC samples, indicating a possible role for MMP24 in the progression of advanced-stage EC [32]. When comparing the relationship between REST and MMP24, MMP24 expression was generally lower than REST levels in the respective cellular localizations; however, an inverse relationship was not observed as previously described in breast cancer cells [18]. Prior studies have shown that when *REST* is knocked down in breast cancer cells, *MMP24* is overexpressed by RNA sequencing [18]. However, it is unclear how low REST levels need to be to see this inverse relationship.

Characterization of *MMP24* gene and MMP24 protein expression in the cell lines did not necessarily correlate. *MMP24* gene expression was higher in Ishikawa compared to t-HESC; however, it was lower in HEC-1-A compared to t-HESC after 24 h of cell growth. MMP24 protein expression trended higher in Ishikawa, but was significantly higher in HEC-1-A compared to t-HESC, indicated by densitometry of the active band at 55 kDa, as previously described [24]. This suggests that transcript levels do not always correlate with protein levels. Also, MMP24 demonstrated multiple bands, indicating the possibility of post-translational modifications or binding to tissue inhibitors of metalloproteinases (TIMPs) [33,34]. Finally, the higher active MMP24 levels in HEC-1-A may parallel the aggressive nature of the cell line and EC sub-type.

To the best of our knowledge, this is the first study to determine REST expression in endometrial cancer and evaluate a potential role for REST as a putative tumor suppressor and prognostic marker in EC. Our study has several strengths. We included a diverse set of patients with at least 18% being minorities in each comparison group. We were also able to investigate REST expression in two different EC cell lines, one thought to be estrogen-driven and the other being a more aggressive sub-type, and found that REST had more of an impact on estrogen-driven cancer. We also evaluated three different functions of tumor spread and metastasis, including proliferation, migration, and invasion to help characterize the role of REST in EC function. Moreover, when evaluating migration and invasion, methods were adopted to inhibit proliferation, which could influence results. MMC was used in the migration assay to prevent cell growth [35]. Further, EC cells were serum starved for 4 h and then harvested in low-CS-FBS (0.1%) media in the invasion assay to stop cell proliferation [31,36,37]. One limitation is the small patient population, which may not accurately reveal important associations between REST and patient demographics or tumor characteristics. Increasing the sample size may be of benefit. Further, we were unable to obtain normal patient tissue specimens adjacent to the tumor in the endometrial cancer samples, which would have helped determine differences in REST expression within a patient depending on tissue morphology. Also, because samples were obtained de-identified, the phase of the menstrual cycle for control patients was unknown, which could have had an impact on REST expression patterns.

Although there are limitations in our study, we have addressed several knowledge gaps related to the relationship between REST and EC. First, we used immunostaining to show the decreased expression of REST human EC samples compared to control samples. We also showed that MMP24 intensity was generally lower than that of REST. We also saw a negative correlation between REST expression in the glandular nuclei and ER percentage in the EC samples, demonstrating a relationship that has previously been identified [18,26]. We further characterized REST expression in EC cell lines compared to a benign cell line. Although REST expression levels were high in the EC cell lines at baseline, decreasing *REST* expression demonstrated an increase in proliferation, migration, and invasion in Ishikawa cells, indicating that REST has a role in the pathogenesis of endometrioid EC.

Some gaps still remain in our knowledge of REST and EC. Questions to consider include determining the association of REST expression levels in patients with EC and the risk of recurrence. Exploring REST and MMP24 levels by immunostaining or RNA sequencing in either normal tissue adjacent to the tumor or in patients with endometrial intraepithelial neoplasia will also help determine the role of these markers in pre-cancerous tissue. Studies determining REST expression in Type II EC patient samples will be interesting as cellular functions were different in Ishikawa cells (Type I EC) and HEC-1-A (Type II EC). Further, we may utilize TCGA data to validate and expand on our findings. In vivo experiments exploring the loss of *REST* in the pathogenesis of EC will also be imperative. Further, most of our experiments have addressed the role and prognostic value of REST in EC; however, identifying therapeutics is crucial since EC is the most common gynecologic malignancy in the United States. As previously mentioned, hormone therapy and its implication for REST levels is of interest given the inverse relationship between REST expression and ER percentage. Also, some studies have identified associations between REST and the phosphoinositide 3 kinase-protein kinase B- mammalian target of rapamycin (PI3K-AKT-mTOR) pathway [38,39]. Preclinical studies investigating inhibitors of this pathway have found moderate response in a variety of cell lines, including some EC cell lines. There are also several sub-classes of drugs that can be used to target this pathway and not all have been studied in EC [40]. Currently, everolimus, an mTOR inhibitor, is approved with the combination of letrozole for the treatment of recurrent endometrioid EC [41,42]. Future translational studies may involve investigating changes in REST expression in EC with PI3K-AKT-mTOR inhibitors. Further, there have been links between REST and histone deacetylases (HDACs) [43]. In particular, HDAC1 and HDAC2 take part in the formation of the CoREST complex, which is a chromatin-modifying co-repressor complex, thought to repress REST and regulate neuronal gene expression and neuronal stem cell fate [43]. There have been preclinical studies investigating HDAC inhibitors and their role in EC; however, they have not addressed the role of REST within this relationship [44,45]. Future studies involving HDAC inhibitors along with their implications for EC and REST expression will be intriguing. Addressing some of these gaps will help in further understanding the role of REST in endometrioid EC.

In conclusion, we showed that REST expression was decreased in EC patient tissue, but increased in EC cell lines. Nevertheless, decreasing *REST* expression in EC cell lines increased proliferation, migration, and invasion, which are hallmarks of cancer progression and metastasis. These findings elicit a potential role for REST as a putative tumor suppressor and prognostic marker in EC. Future experiments will involve in vivo studies exploring the loss of *REST* in the pathogenesis of EC. Further, investigating treatment effects on REST and EC with hormone therapy, PI3K-AKT-mTOR inhibitors, and/or HDAC inhibitors will be essential to using REST as a prognostic marker.

## 4. Materials and Methods

### 4.1. Human Subjects and Tissue Acquisition

Deidentified human tissue samples were obtained from the University of Kansas Cancer Center’s Biospecimen Repository Core Facility (BRCF), which were collected between 2010 and 2023. Formalin-fixed paraffin-embedded tissues including the endometrium were cut and mounted (5 µm) and acquired as unstained slides. Controls (*n* = 16) included patients undergoing hysterectomy for benign indications. Patients with a history of leiomyoma and adenomyosis were excluded due to the possibility of differential expression of REST in these tissues. Samples were also obtained from patients undergoing hysterectomy for endometrioid EC or Type I EC, including early-stage (N = 15) and advanced-stage (N = 16). Patients with other histologic sub-types of EC were excluded. Additional patient characteristics were attained including age, body mass index (BMI) in kg/m^2^, race, comorbidities including diabetes, hypertension, and/or hyperlipidemia, medications for these illnesses, and smoking and alcohol use history. For patients with EC, tumor characteristics were abstracted including grade, presence of LVSI, estrogen and progesterone receptor (ER/PR) percentage, presence or absence of abnormal TP53, and the status of MMR proteins including MLH1, PMS2, MSH2, and MSH6.

### 4.2. Immunohistochemistry

Formalin-fixed paraffin-embedded tissues from control and EC patients from the BRCF were used for immunohistochemistry to detect the expression of REST (1:1000; Proteintech, Rosemont, IL, #22242-1-AP) and MMP24 (1:250; Invitrogen/Thermo Fisher Scientific, Waltham, MA, USA, #PA5-119273). Tissue sections were deparaffinized. Antigen retrieval was carried out with slides submerged in citric acid-based solution (pH 6.0) per the manufacturer’s instructions (Vector Laboratories, Inc., Burlingame, CA, USA, #H-3300) at 95 °C for 10 min. IHC was performed using the Vectastain ABC Kit (Vector Laboratories, #PK-6106). Protein localization was performed with the ImmPACT DAB Substrate Kit (Vector Laboratories, #SK-4105) and was identified as brown staining. Sections were counterstained with hematoxylin (Vector Laboratories, #H-3404) and cover-slipped with mounting media. Images were taken on the Nikon Eclipse 80i Upright microscope (Melville, NY, USA).

Antibody intensity was quantified using H-scoring in the nuclei and cytoplasm of the endometrial glands and stroma [46,47]. Briefly, a score of 0 indicated absence of staining, 1 indicated minimal staining, 2 indicated moderate staining, and 3 indicated strong staining. Two independent observers analyzed the slides for H-scoring and values were averaged.

### 4.3. Cell Culture and Cell Transfection

Two human EC cell lines were used, including the Ishikawa cell line, which is a Type I EC [31] that was provided by Dr. Bruce Lessey (Wake Forest University School of Medicine, Winston-Salem, NC, USA), and the HEC-1-A cell line (ATCC HTB112™), which is a Type II EC [31] that was purchased from the American Type Culture Collection (ATCC; Manassas, VA, USA). The human endometrial stromal cell line (t-HESC, ATCC CRL4003^TM^) was also obtained from the ATCC and used as a control.

Cells were cultured in Dulbecco’s Modified Eagle’s Medium (DMEM)/Ham’s F-12 50/50 Mix with L-glutamine (Corning; Corning, NY, USA) with 10% Fetal Bovine Serum (FBS; Atlanta Biologicals, Atlanta, GA, USA), 1% penicillin-streptomycin (Pen Strep; Life Technologies, Carlsbad, CA, USA), and 2 µL/mL of normocin (InvivoGen, San Diego, CA, USA) in T75 flasks until approximately 90% confluent. All cells were grown in a humidified incubator under standard culture conditions of 21% O_2_ and 5% CO_2_ at 37 °C. Cells were passaged and then plated for mRNA and protein isolation. Cells were also transfected for subsequent functional assays. To do so, once cells reached near confluency, they were trypsinized and resuspended in DMEM with 2% charcoal stripped (CS)-FBS (Atlanta Biologicals, Atlanta, GA, USA) with 1% Pen-Strep and 2 µL/mL normocin and prepared at the desired density in a total volume of 1.5mL of the media above and transfection reagents for a 6-well plate.

Then, EC cell lines were separately transfected, as previously described [46]. Briefly, in order to decrease the RNA expression levels of *REST*, siRNA to *REST* (ON-TARGETplus SMARTPool; L-006466-00-0020; GE Healthcare Dharmacon, Inc.; Lafayette, CO, USA) was used and brought to a final concentration of 50nM. A non-targeting (*NT*) mimic (ON-TARGETplus Control; D-001810-10-50 GE Healthcare Dharmacon, Inc.; Lafayette, CO, USA) was used as a control and was also prepared similarly. siRNAs were individually incubated and then combined with siPORT™ NeoFX™ transfection agent (Invitrogen/Thermo Fisher Scientific, Waltham, MA, USA) in DMEM without FBS and 200uL of each reagent was added to a 6-well plate, individually. The cell mixture was then added to bring each well to a final volume of 1.5mL. Due to the high expression levels of *REST* (unpublished observation), a second transfection was conducted 24 h after the initial reverse transfection using Lipofectamine-2000 (Invitrogen/Thermo Fisher Scientific, Waltham, MA, USA). Similarly, siRNA to *REST* and *NT* were individually incubated with Lipofectamine-2000 for 10 min. Media were removed from each well and replaced with DMEM with 2% CS-FBS. *NT* or *REST* siRNA was added to each corresponding well. Twenty-four hours after the second transfection, the impact of *REST* depletion on cellular proliferation, migration, and invasion was assessed. To confirm that *REST* gene expression was decreased, qRT-PCR was performed as described below.

### 4.4. RNA Isolation and qRT-PCR

Non-transfected cells were plated at a density of 3.0 × 10^5^ cells/well in a 6-well plate and allowed to attach and divide to near confluency. Total RNA was isolated using TRIzol reagent (Invitrogen/Thermo Fisher Scientific, Waltham, MA, USA) after 24 and 48 h. The pellet was dried and resuspended in 30 µL of nuclease-free water. RNA concentrations were measured using the NanoDrop 1000 Spectrophotometer (Thermo Fisher Scientific, Waltham, MA, USA). Then, reverse transcription (RT) was performed using 1 µg in 20 µL with the M-MLV Reverse Transcriptase kit (Invitrogen/Thermo Fisher Scientific, Waltham, MA, USA) and Random Primers (Invitrogen/Thermo Fisher Scientific, Waltham, MA, USA).

Relative gene expression was determined by qRT-PCR on a QuantStudio 7 Flex System (Applied Biosystems/Thermo Fisher Scientific, Foster City, CA, USA) for EC cell lines normalized to the t-HESC line. The relative expression of *REST* (*REST* TaqMan Gene Expression Assay (FAM), Life Technologies/Thermo Fisher Scientific, Carlsbad, CA, USA, #4331182) was quantified by fold change (2−ΔΔCt) as compared to *β-Actin* (*ACTB*; Integrated DNA Technologies, Coralville, Iowa, USA; Forward 5′-GCACAGAGCCTCGCCTTT-3′, Reverse 5′-TATCATCATCCATGGTGAGCTGG-3′) as the housekeeping gene. *REST* primer was prepared in TaqMan Universal Master Mix No AmpErase UNG (Applied Biosystems/Thermo Fisher Scientific, Foster City, CA, USA) and *ACTB* was prepared with PowerSYBR Green PCR Master Mix (Applied Biosystems). All samples were run in triplicate for N = 4 at two different time points (24 and 48 h).

### 4.5. Protein Isolation, Western Blotting, and Quantification

Non-transfected cells were plated at a density of 1.5 × 10^6^ cells/plate in 10 cm plates and allowed to attach and divide to near confluency. Whole-cell lysates were collected from Ishikawa, HEC-1-A, and t-HESC cell lines using cell lysis buffer (Cell Signaling Technology, Danvers, MA, USA) with 1mM PMSF (Cell Signaling Technology) after 24 and 48 h. A Bio-Rad Protein Assay (Bio-Rad Laboratories, Richmond, CA, USA) was used to determine the protein concentration with bovine serum albumin (BSA) as standard using the Pharmacia Biotech Ultrospec 2000 UV spectrophotometer.

For each cell line, 30 µg of protein was denatured and fractionated by electrophoresis using Precast Mini-PROTEAN TGX Gels with a 4–15% gradient gel (Bio-Rad Laboratories) and electro-blotted onto a nitrocellulose membrane (Invitrogen/Thermo Fisher Scientific, Waltham, MA, USA). Primary antibodies REST (1:2000; Proteintech) and MMP24 (1:500; Invitrogen/Thermo Fisher Scientific, Waltham, MA, USA) and the secondary antibody donkey anti-rabbit IgG-Horseradish Peroxidase (HRP)-linked (1:5000; Cytiva, Marlborough, MA, USA #NA934) were used. To normalize the protein expression levels, the membranes were stripped and re-probed for β-tubulin (1:1000; Cell Signaling Technology, #2146). The immuno-detection was performed by using an enhanced chemiluminescence (ECL) kit (Thermo Fisher Scientific, Waltham, MA, USA).

REST bands were quantified and normalized to β-tubulin bands using ImageJ Fiji, Version 2.1.0/1.53c [48] and EC cell lines were compared to t-HESC. All samples were run for N = 3 at two different time points. MMP24 bands were analyzed in a similar manner for N = 2 at two different time points.

### 4.6. Cellular Proliferation

Each EC cell line was double transfected with either *NT* or *REST* siRNA with a density of 1.5 × 10^5^ cells/well for the cellular proliferation assay using crystal violet, as previously described [46]. A lower density was used to allow an area for cells to proliferate. Briefly, cells were cultured for a total of 48 h with transfection agents and media were aspirated. Each well was rinsed with phosphate-buffered solution (PBS). Crystal violet staining solution (0.125 g crystal violet powder in 50 mL of 20% (*v*/*v*) methanol) was added to each well for 10 min at room temperature (RT). The staining solution was removed and wells were rinsed with 1X PBS six times. Lysis buffer (0.1 M sodium citrate, 50% ethanol (*v*/*v*), pH 4.2) was then added and incubated for 30 min at RT on a shaker. The lysate from each well was then diluted 1:20 with deionized H_2_O and read as optical density (OD) at 590 nm on the Pharmacia Biotech Ultrospec 2000 UV spectrophotometer in duplicate and values were averaged. The assay was performed in triplicate for *NT* and *REST* siRNA-transfected cells for a total N = 3 and each experiment resulted from a different cell passage number. Data were normalized to the *NT* siRNA samples and experimental data were combined.

### 4.7. Cell Migration Assay

In a similar fashion, each cell line was double transfected with either *NT* or *REST* siRNA, but using a density of 3.0 × 10^5^ cells/well and allowed to attach and divide to near confluency. Scratch assays were performed as previously described with few modifications [46]. Briefly, cells were cultured to near confluency and 2.5 µg/mL of mitomycin C (MMC; Sigma Life Sciences, Burlington, MA, US) was added for 2 h to halt proliferation [35]. MMC was removed and a wound was created using a p1000 pipette tip in the midline of each well. Wells were rinsed with 1X PBS once and DMEM with 2% CS-FBS media was replaced. Images were taken with the Olympus IX71 Inverted microscope at 0 h, 24 h, and 48 h and the area within a designated part of the well was measured using ImageJ Fiji, Version 2.1.0/1.53c [48]. Migration was measured as the change in area over time. The wound closure percentage was also reported as the change in area over the area at time 0 h × 100%. The assay was performed in triplicate for *NT* and *REST* siRNA-transfected cells for a total N = 3. Each experiment resulted from a different cell passage number. Data were normalized to the *NT* control and experimental data were combined.

### 4.8. Trans-Well Cellular Invasion Assay

Trans-well invasion assays were performed using 24-well plates (Corning, #353504) and cell culture inserts with 8 µm sized pores (Corning, #353097) as previously described [47,49]. Briefly, cell culture inserts were coated with phenol red-free, growth-factor-reduced Matrigel™ (Corning, #356231) mixed with FBS-free DMEM at a final concentration of 0.3 mg/mL for a thick coating and incubated for 4 h in 37 °C. EC cells were serum starved in FBS-free DMEM for 4 h before resuspension in DMEM with 0.1% CS-FBS. DMEM with 10% CS-FBS was used as a chemoattractant in the lower compartment of the well. *NT* or *REST* siRNA mixed with siPORT™ NeoFX™ transfection agent was added to each corresponding insert along with 2.0 × 10^5^ cells in a total volume of 400 µL media. Cells were allowed to attach to the membrane and after 24 h, media were removed and replaced with *NT* or *REST* siRNA mixed with Lipofectamine-2000 for an additional 24 h. After this time, invading cells on the undersurface of the membrane were fixed with 4% paraformaldehyde (PFA) and rinsed with PBS. The membrane was excised with a scalpel, mounted on a glass slide with DAPI (4′,6-diamidino-2-phenylindole; Vector Laboratories) and cover-slipped. Slides were imaged using the Olympus IX71 Inverted Microscope. Images were analyzed with ImageJ Fiji [48]. Percent invasion was represented as number of cells invading through the Matrigel™ membrane over an area of the membrane [47,49,50]. The assay was performed in triplicate for N = 3. Each assay was performed using a different cell passage number and experimental data were combined after normalizing to the *NT* control.

### 4.9. Statistical Analysis

Statistical analysis was performed using GraphPad Prism Version 9.2.0 (Boston, MA, USA). For human IHC analysis, data were evaluated for normalcy of distribution using the Kolmogorov–Smirnov or Shapiro–Wilk test, where appropriate. For patient groupwise comparisons, the Kruskal–Wallis test was used for continuous variables and Chi-Square test was used for categorical data. Comparisons of REST expression among controls, early-stage, and advanced-stage EC specimens in each localization were carried out using the Kruskal–Wallis test. Further, a univariate analysis with multiple linear regression was performed with REST expression as the dependent variable and age, body mass index (BMI) in kg/m^2^, comorbidities including diabetes, hypertension, and/or hyperlipidemia, medications for these illnesses, and smoking and alcohol use history as independent variables. Multiple linear regression was also performed on cancer specimens investigating the relationship among REST expression and tumor characteristics. Correlations were also assessed using Spearman’s correlation coefficient. Further, the relationship between REST and MMP24 expression in each localization was investigated using the Wilcoxon Rank test.

For cell line studies, data were evaluated for normalcy of distribution as above. Baseline *REST* and *MMP24* gene expression and protein expression in EC cell lines was compared to that in the t-HESC line using the parametric *t*-test or nonparametric Mann–Whitney-U test, as appropriate. Similar methods were used for comparisons in proliferation, migration, and invasion between *NT* or *REST* siRNA samples.

Data are expressed as medians with interquartile range (IQR) for nonparametric data and means with standard deviation (SD) for parametric data or frequency (percentage), as appropriate. Data comparisons are reported as fold change (FC). Data are represented as the β-coefficient with a 95% confidence interval (CI) for linear regression studies. Significance was set at *p* < 0.05 for all experiments.

## Figures and Tables

**Table 1:** Baseline demographics.

	Controls (N = 16)	Early-Stage EC (N = 15)	Advanced-Stage EC (N = 16)	*p*-Value
Age	62 (6, 73)	63 (53, 72)	62 (57, 66)	*p* = 0.85
BMI	31.0 (25.0, 38.4)	30.8 (26.7, 35.0)	34.6 (27.0, 42.2)	*p* = 0.58
Race				
White	13 (81.3)	8 (53.3)	9 (56.3)	*p* = 0.50
Black	2 (12.5)	4 (26.7)	2 (12.5)	
Asian	0 (0)	1 (6.7)	1 (6.2)	
Other	1 (6.2)	2 (13.3)	4 (25.0)	
Comorbidities				
Diabetes	2 (12.5)	6 (40.0)	8 (50.0)	*p* = 0.07
Hypertension	8 (50.0)	10 (66.7)	9 (56.3)	*p* = 0.61
Hyperlipidemia	9 (56.3)	10 (66.7)	8 (50.0)	*p* = 0.64
Medications				
Anti-diabetes	2 (12.5)	4 (26.7)	7 (43.8)	*p* = 0.14
Anti-hypertensives	7 (43.8)	5 (33.3)	4 (25.0)	*p* = 0.53
Anti-lipid	5 (31.3)	6 (40.0)	6 (37.5)	*p* = 0.91
Smoking History	5 (31.3)	6 (40.0)	5 (31.3)	*p* = 0.84
Alcohol History	6 (37.5)	6 (40.0)	7 (43.8)	*p* = 0.94

Data are represented as median with [IQR] or frequency (%). *p* < 0.05 was considered significant.

**Table 2:** Tumor characteristics.

	Early Stage (N = 15)	Advanced Stage (N = 16)	*p*-Value
Stage			
1	10 (66.7)	(0)	
2	5 (33.3)	(0)	
3	0 (0)	16 (100.0)	
4	0 (0)	0 (0)	
Grade			
1	11 (73.3)	6 (37.5)	*p* = 0.02
2	1 (6.7)	8 (50.0)	
3	3 (20.0)	2 (12.5)	
ER	90 (80, 98)	83 (82, 99)	*p* = 0.75
PR	80 (50, 97)	79 (23, 90)	*p* = 0.33
p53	1 (6.7)	0 (0.0)	*p* = 0.48
dMMR	3 (20.0)	5 (31.3)	*p* = 0.69

Data are represented as frequency (%). *p* < 0.05 was considered significant.

## Data Availability

All data are contained within the article.

## Supplementary Materials

The following supporting information can be downloaded at: https://www.mdpi.com/article/10.3390/ijms25179693/s1, Figure S1. Western blot images of REST protein expression in cell lines at 24 h, Figure S2. Western blot images of REST protein expression in cell lines at 48 h, Figure S3. Western blot images of MMP24 protein expression in cell lines at 24 h, Figure S4. Western blot images of MMP24 protein expression in cell lines at 48 h.
