# Supplementary material for: Functional Analysis of RE1 Silencing Transcription Factor as a Putative Tumor Suppressor in Human Endometrial Cancer
Source: Int J Mol Sci. 2024 Sep 7;25(17):9693. doi: 10.3390/ijms25179693 (PMC11395688; doi:10.3390/ijms25179693)
Supplement: Supplementary file 1 [file ijms-25-09693-s001.zip › ijms-3168771-supplementary.pdf]

Supplementary Figures

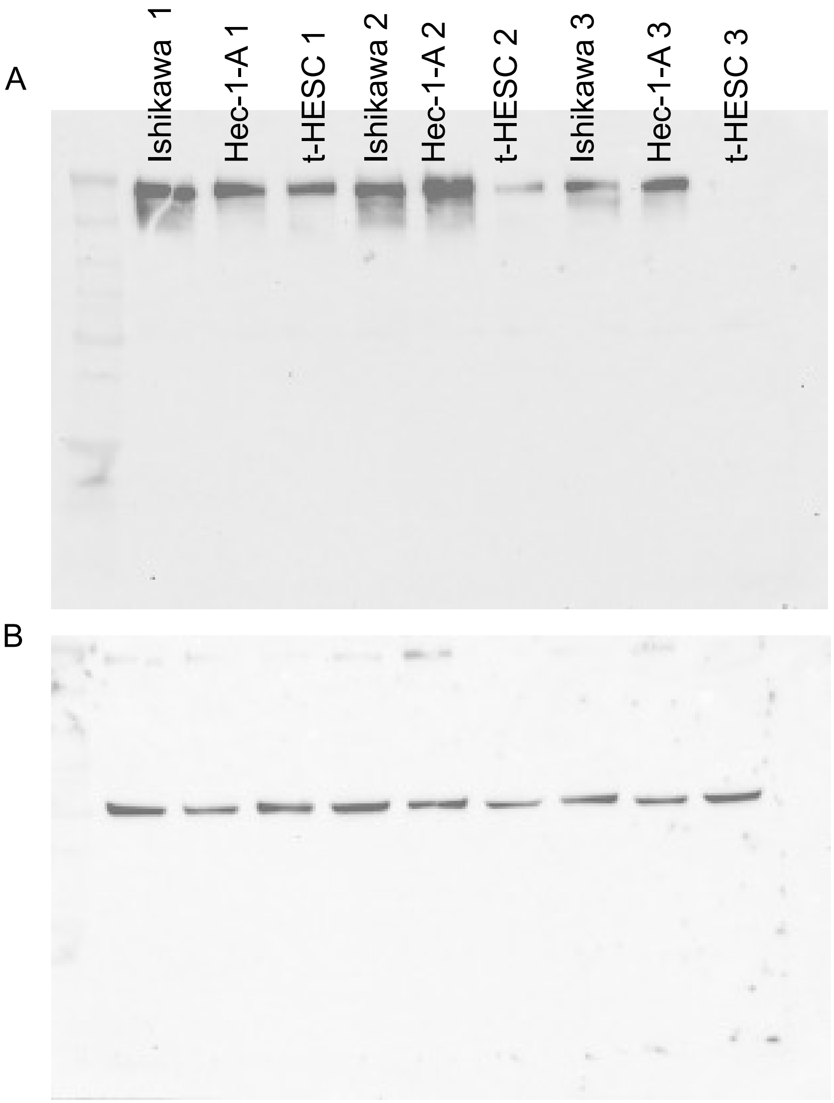

Antibody: REST
Proteintech 22242-1-AP
Dilution: 1: 2000
Size: 200k Da

Antibody: β-tubulin
Cell signaling technologies 2146
Dilution: 1:1000
Size: 55 kDa

Figure S1. Western blot images of REST protein expression in cell lines at 24 hours. A. REST protein expression at 24h **B**. β-tubulin as the sample processing control at 24h

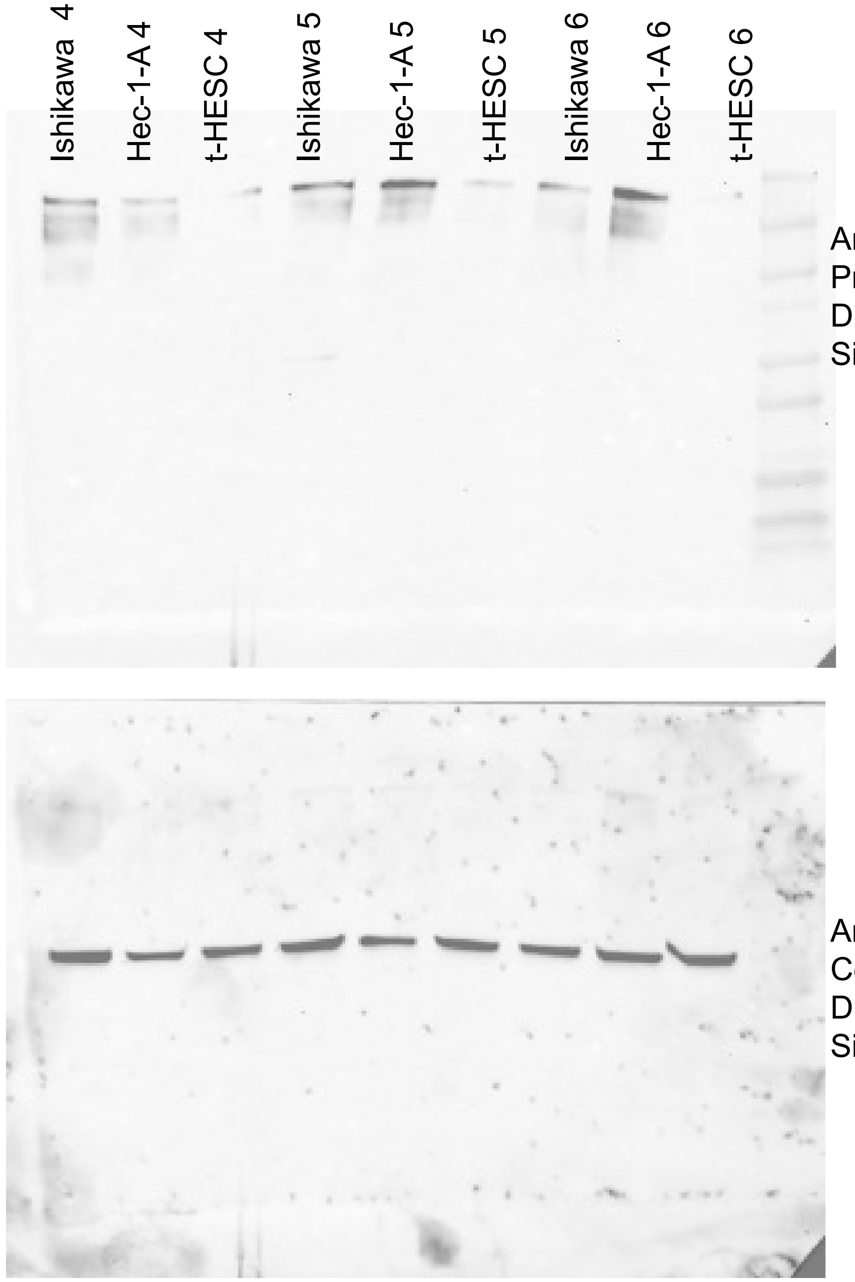

A

Antibody: REST
Proteintech 22242-1-AP
Dilution: 1: 2000
Size: 200 kDa

B

Antibody: β-tubulin
Cell signaling technologies 2146
Dilution: 1:1000
Size: 55 kDa

Figure S2. Western blot images of REST protein expression in cell lines at 48 hours. A. REST protein expression at 48h **B**. β-tubulin as the sample processing control at 48h

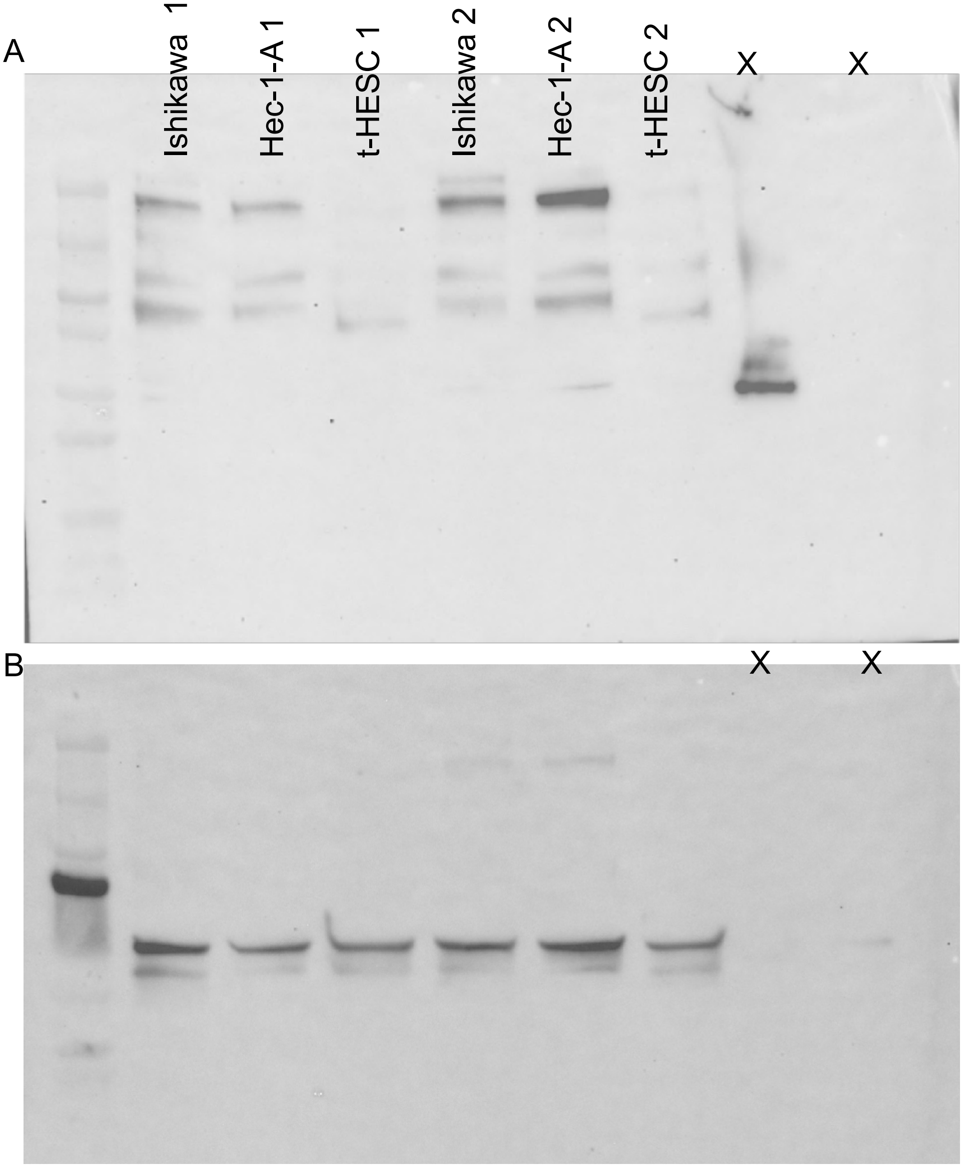

Antibody: MMP24
Invitrogen: PA5_119273
Dilution: 1:500
Size: 73 kDa, 55 kDa

Antibody: β-tubulin
Cell signaling technology 2146
Dilution: 1:1000
Size: 55 kDa

Antibody: β-actin
Invitrogen: ab8227
Dilution: 1:5000
Size:42 kDa

Figure S3. Western blot images of MMP24 protein expression in cell lines at 24 hours. A. MMP24 protein expression at 24h **B**. β-tubulin as the sample processing control at 24h

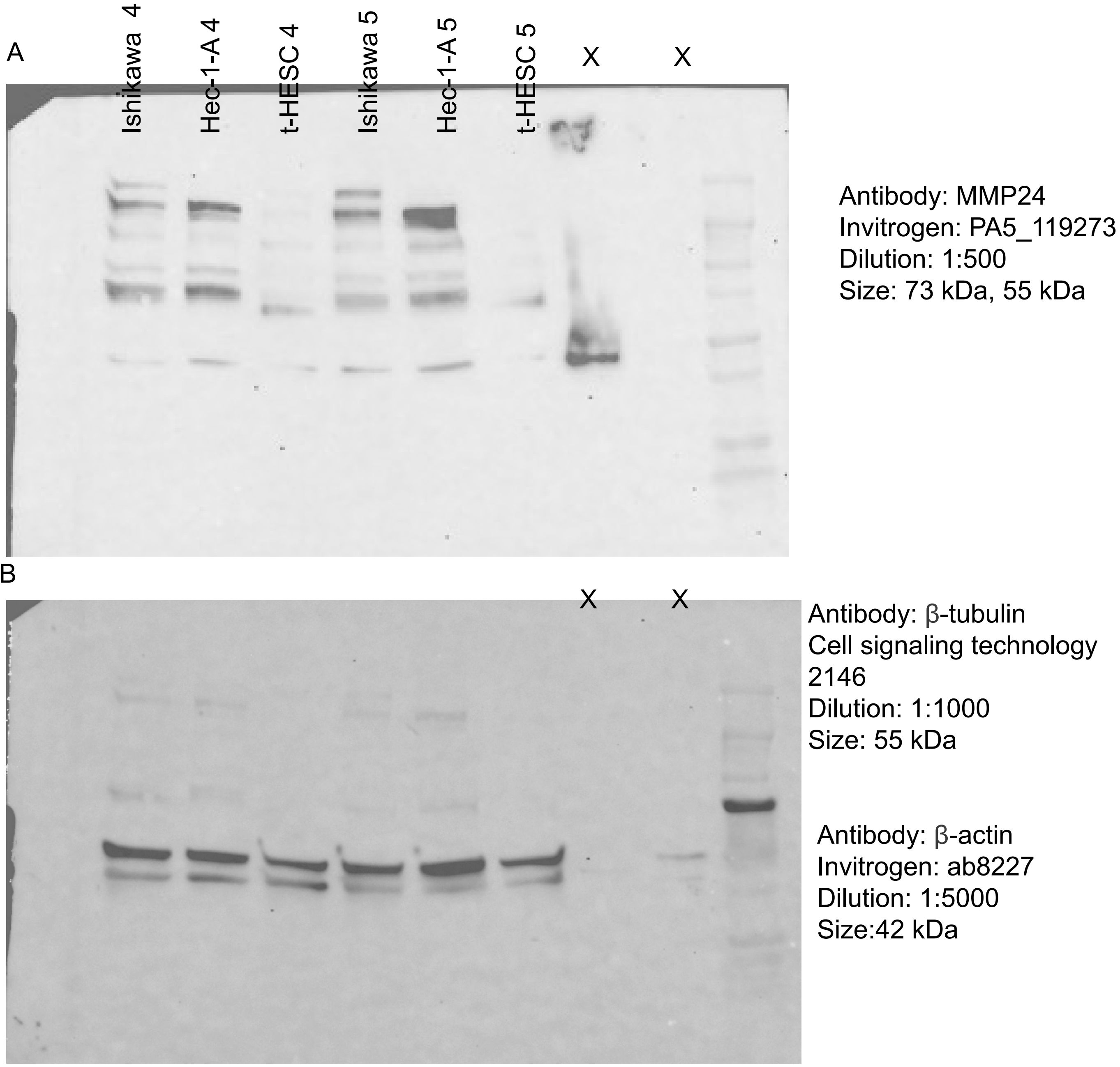

Figure S4. Western blot images of MMP24 protein expression in cell lines at 48 hours. A. MMP24 protein expression at 48h B. β-tubulin as the sample processing control at 48h
